# Maternal depressive symptoms and young people's higher education participation and choice of university: Evidence from a longitudinal cohort study

**DOI:** 10.1016/j.jad.2023.10.061

**Published:** 2023-10-16

**Authors:** Sally Bowman, Tim T. Morris, Matt Dickson, Frances Rice, Laura D. Howe, Amanda M. Hughes

**Affiliations:** a Avon & Wiltshire Mental Health Partnership NHS Trust, Bath, UK; b Centre for Longitudinal Studies, Social Research Institute, University College London, London, UK; c Institute for Policy Research, University of Bath, Bristol, UK; d Wolfson Centre for Young People's Mental Health, Division of Psychological Medicine and Clinical Neurosciences, School of Medicine, Cardiff University, Bristol, UK; e Population Health Sciences, University of Bristol, Bristol, UK; f MRC Integrative Epidemiology Unit, University of Bristol, Bristol, UK

**Keywords:** Maternal depression, Educational attainment, Higher education, Locus of control, Intergenerational, ALSPAC

## Abstract

**Background:**

Participation in higher education has significant and long-lasting consequences for people's socio-economic trajectories. Maternal depression is linked to poorer educational achievement for children in school, but its impact on university attendance is unclear.

**Methods:**

In an English longitudinal cohort study (*N* = 8952), we explore whether young people whose mothers experienced elevated depressive symptoms are less likely to attend university, and the role of potential mediators in the young person: educational achievement in school, depressive symptoms, and locus of control. We also examine whether maternal depressive symptoms influence young people's choice of university, and non-attendees’ reasons for not participating in higher education.

**Results:**

Young people whose mothers experienced more recurrent depressive symptoms were less likely to attend university (OR = 0.88, CI = 0.82,0.94, *p* < 0.001) per occasion of elevated maternal depressive symptoms) after adjusting for confounders. Mediation analysis indicated this was largely explained by educational achievement in school (e.g., 82.7 % mediated by age 16 achievement) and locus of control at 16. There was mixed evidence for an impact on choice of university. For participants who did not study at university, maternal depressive symptoms were linked to stating as a reason having had other priorities to do with family or children (OR: 1.17, CI = 1.02,1.35).

**Limitations:**

Lack of data on the other parent's depression, loss to follow-up, possibly selective non-response.

**Conclusions:**

Young people whose mothers experience elevated depressive symptoms on multiple occasions are less likely to participate in higher education; educational achievement in secondary school, but not the young people's own depressive symptoms, substantially mediated the effect.

## 1 Introduction

Participation in higher education has significant and long-lasting consequences for young people's socioeconomic trajectories (Britton et al., 2020). Compared to people without a degree, individuals with an undergraduate degree earn more both early in their careers (Belfield et al., 2020) and over their lifetime (Britton et al., 2020). Moreover, because graduates are more likely than non-graduates to have a parent who attended university, participation in higher education is thought to contribute to the intergenerational transmission of inequality (Black and Devereux, 2011; Eyles et al., 2022). In England, just over half of young people currently participate in higher education(Department for Education, 2023).

One factor which may affect whether a young person studies at higher education is maternal depression. In the UK, depression is estimated to affect around 1 in 5 mothers with children under the age of 16 (Abel et al., 2019). Since this only considers recorded diagnoses in primary care, the true prevalence is likely higher. Existing studies suggest maternal depression can have significant impacts on young people's mental health and socioemotional development (Goodman et al., 2011), and educational attainment throughout childhood and adolescence. Young people whose mothers had prenatal or postnatal depression have lower educational attainment throughout schooling (Psychogiou et al., 2020) and are less likely to achieve passing grades in General Certificate of Secondary Education (GCSE) exams, usually taken at age 16 (Pearson et al., 2016). This may reduce opportunities for higher education participation because university entry requirements often include specific GCSE grades in key subjects.

Maternal depression could be linked to young people's participation in university for several reasons. First, observed associations between maternal depression and children's attainment may be due to common factors influencing the parent and the child. For example, maternal depression is strongly associated with socioeconomic deprivation (Abel et al., 2019) which may independently impact young people's mental health (Melchior et al., 2010; Zaneva et al., 2022) and educational attainment (Stopforth et al., 2021). Alternatively, socioeconomic factors could mediate rather than confound associations: maternal depression may impact on a family's income (Thomson et al., 2022), which could in turn influence children's attainment. Third, associations between maternal depression and children's attainment could be driven by depression in the young person. Depression often runs in families, with children of depressed parents 3–4 times more likely to suffer from depressive disorder than controls (Rice et al., 2002). At the same time, adolescent depression is likely to negatively impact educational outcomes (López-López et al., 2021; Wickersham et al., 2021). Meanwhile, there may be protective influences of cognitive attitudes such as locus of control and self-efficacy (Buddelmeyer and Powdthavee, 2016; Collishaw et al., 2016; Ng-Knight and Schoon, 2017). Locus of control refers to how much a person believes events in their life are within their control, rather than being a function of chance, fate, or under the control of others (Rotter, 1966). A more ‘internal’ locus of control denotes greater perceived control over these events or agency over one's own life. Previous studies suggest a more internal locus of control may protect against psychological and socioeconomic effects of adversity (Buddelmeyer and Powdthavee, 2016; Ng-Knight and Schoon, 2017). Self-efficacy describes an individual's perceived ability to overcome problems, cope with adversity and achieve difficult tasks (Schwarzer et al., 1995), and adolescents with high self-efficacy are less likely to develop mood and behaviour issues even if their parents had depression (Collishaw et al., 2016).

Besides higher education participation, maternal depression may influence the young person's choice of university. Young people in the UK typically move away from their hometown to attend university in a new city or town, and perceived distance from home may affect a young person's adjustment to university (Mooney et al., 1991). Those whose mothers have experienced or continue to experience depression may decide to stay closer to home to more easily provide support if needed. This could lead to ‘undermatching’, when a student chooses a course or institution whose entry requirements are lower than their attained grades, which can negatively impact a student's prospects (Dillon and Smith, 2020). Since undermatching is more common for less socioeconomically advantaged students (Campbell et al., 2021), it may also contribute to intergenerational transmission of (dis)advantage.

This study explores the impact of maternal depressive symptoms during a young person's lifetime on higher education participation, the distance travelled to university, and the reasons for these choices, using longitudinal data from an English birth cohort study. Existing studies have focused on maternal depression or depressive symptoms in the pre- and postnatal periods, examining their impact on children's educational attainment until age 16 (Pearson et al., 2016; Psychogiou et al., 2020). We consider maternal depressive symptoms from when the young person is one year old until they are 18 years old, the typical age of entry to higher education in the UK, to examine their impact on later educational outcomes. In a novel contribution, we use longitudinal data to investigate possible mediators of associations, considering the young person's own depressive symptoms, prior educational attainment, and locus of control in adolescence. We explore young people's own accounts of their reasons for attending a particular university, or for not attending university at all. Finally, we investigate whether young people affected by maternal depression attend universities which are closer to home, which could indicate undermatching in this group.

## 2 Methods

### 2.1 Participants

This analysis used data from the Avon Longitudinal Study of Parents and Children (ALSPAC), a pregnancy study of women living in or around Bristol with expected delivery dates between 1/4/1991 and 31/12/1992 (Boyd et al., 2013; Fraser et al., 2013; Northstone et al., 2019). From the initial 14,541 pregnancies, 13,988 children were alive at 1 year (for more details see Supplementary Methods).

We used data from questionnaires completed by mothers during pregnancy and when the young person was 1, 5, 8, 11 and 18 years old. We also used data collected from young people via questionnaires when they were 16, 18 and 26 years old. The study website contains details of all the data that is available through a fully searchable data dictionary and variable search tool: http://www.bristol.ac.uk/alspac/researchers/our-data/. Information on young people's educational achievement at age 14 and 16 was obtained through data linkage of ALSPAC to the National Pupil Database (NPD), an administrative database which represents the most accurate and extensive record of individual educational achievement available in England. Pupils in state schools are routinely included but young people in some private schools or those who are home-educated are not (Jay et al., 2019).

Analyses were restricted to young people who had linked data on educational achievement at 14 or 16 and where the mother had completed a measure of depressive symptoms at least once during the child's lifetime (*N* = 8952). A STROBE diagram showing exclusions from the analytic sample is provided in the supplementary material (Fig. S1). Analysis specific to university attendees (of the distance travelled to university, and reasons for choosing that institution) was further restricted to young people who had completed the age 26 questionnaire, reporting that they had studied at university (*N* = 2037). Analysis specific to non-attendees (of reasons for not going to university) was restricted to young people who had completed the age 26 questionnaire, reporting that they had not studied at university (*N* = 1129).

### 2.2 Measures

#### 2.2.1 Maternal depressive symptoms

Maternal depressive symptoms were measured using the Edinburgh Postnatal Depression Scale (EPDS) when the young person was aged one, 5, 8, 11, and 18 years old. This scale is typically used within the postnatal period but has been validated for use within mothers past this period (Cox et al., 1996). The EPDS includes 10 items covering the individual's emotional status over the previous week and is scored between 0 and 30 with higher scores indicating greater depressive symptoms. The main exposure was a continuous measure which considered how many times, during the young person's childhood and adolescence, the mother scored 13 or more on the EDPS. This cut-off has been shown to have a high level of sensitivity in detecting clinically relevant depression in mothers (Cox et al., 1996). Using all available EPDS measurements in this period, we constructed a measure capturing symptoms at multiple time points, as recurring parental depression may be especially linked to young people's outcomes (Collishaw et al., 2016). In a sensitivity analysis, we examined associations with the mother's raw EPDS score when the young person was aged 11, the last available exposure measurement preceding the mediators (all measured between age 14 and age 16). It is also the age of transition to secondary school, which evidence suggests is important for later educational outcomes (West et al., 2010). We also examined associations with the mother's raw EPDS score when the young person was aged 18 (when young people in the UK typically leave for university).

#### 2.2.2 Higher education participation

The main outcome was whether the young person studied at higher education. In a questionnaire completed at age 26, participants were asked whether they had studied at university for a degree or higher qualification. This was coded as yes/no, by grouping young people who were currently at university with those who had attended into the past.

The Euclidian distance in kilometres between a participant's home at 18 and their university was calculated based on home postcodes and self-reported information on the institution attended from the age 26 questionnaire. The overwhelming majority of observations (99.4 %) were 617 km or less, consistent with distances within the UK. For fifteen participants, distances were between 1074 km and 17,063 km, and were recoded to 617 km to limit the influence of outliers who had moved abroad.

Also in the age 26 questionnaire, participants who had attended university reported why they had chosen that institution, rating a set of considerations from 1 “extremely unimportant” to 5 “extremely important”. This analysis focused on two considerations that a priori we considered most relevant to this research question: “university's distance to family home” and “had to stay close to family/children”. Meanwhile, participants who had not attended university reported their reasons for this choice. Again, they were asked to rate a set of possible considerations from 1 “extremely unimportant” to 5 “extremely important”. We focused on two reasons selected a priori based on potential relevance: “I didn’t want to be financial burden” and “I had other priorities (e.g., family/children)”. All reasons were recoded to important or not important (by collapsing, respectively, important and extremely important, and extremely unimportant to neither important nor unimportant).

#### 2.2.3 Mediators

The young person's own depressive symptoms at age 16 were measured with the Short Moods and Feelings Questionnaire (SMFQ) (Thabrew et al., 2018). This was the last available measure before young people apply for university, typically around age 17. The SMFQ contains 13 items assessing depressive symptoms over the previous two weeks. This scale has been shown to have a high level of validity in detecting mild to moderate depression in adolescents (Thabrew et al., 2018). Total scores for the SMFQ range between 0 and 26 with higher scores indicating greater depressive symptoms.

We also considered mediation by the young person's educational achievement at ages 14 and 16, using summary scores derived from linked educational data from the NPD (Jay et al., 2019). For age 14 educational achievement, we used a summary score based on test results from English, Maths and Science, a continuous measure with a possible range of 0–141. Age 16 educational achievement was based on General Certificate of Secondary Education (GCSE) qualifications sat at the end of compulsory schooling. We used the capped GCSE and equivalents points score, a continuous measure based on a pupil's best eight subjects with a possible range of 0–540 (Department for Education, 2015).

Lastly, we considered the young person's locus of control at age 16, measured using a 12-item version of the Children's Nowicki-Strickland Internal-External scale (CNSIE) (Nowicki and Strickland, 1973). This is based on the young person's responses to questions such as “When nice things happen to you is it usually because of ‘luck’?” and “Do you think that preparing for things is a waste of time?”. Lower scores indicate an internal locus of control, meaning the person is more likely to believe their own behaviours can influence the outcomes of events, while higher scores indicate an external locus of control.

#### 2.2.4 Confounders

Confounders included the young person's biological sex at birth (male/female) and the mother's age at the child's birth (in years). We included as confounders two aspects of maternal socioeconomic position: educational attainment and occupational social class. Compared to income-based measures, these are relatively stable in adult life (Galobardes et al., 2007), and therefore less likely to be influenced by maternal depressive symptoms during the child's lifetime. Both were self-reported at 32 weeks gestation. Highest qualification was categorized as: none, GCSEs or O-levels (subject-based qualifications usually taken at age 16), A-levels (academic qualifications usually taken at age 18), vocational qualifications, and university degree or higher. Occupational social class was based on the Registrar General's Social Classification (Last, 2007). This was categorized as: professional, managerial, skilled non-manual, skilled manual, semi-skilled, and unskilled occupations. We also controlled for whether the mother reported taking part in employment or voluntary work when the young person was 11 years old (coded as yes/no).

### 2.3 Statistical analysis

Within the analytic sample (*N* = 8952), we conducted multiple imputation with chained equations to impute missing data (m = 50). Three sets of imputation models were run. The first included all 8952 participants and was used for analysis of higher education participation. The second was restricted to the 2037 participants who at age 26 reported that they had attended university; this imputed dataset was used for analysis of outcomes specific to university attendees. The third was restricted to the 1139 who at age 26 reported that they had not attended university; this was used for analysis of outcomes specific to non-attendees. Details of the imputation procedure are provided in Supplementary Methods, and the proportion of imputed data for each variable in Supplementary Table S1.

Within all 8952 young people in the analytic sample, logistic regression was used to examine associations between maternal depressive symptoms and higher education participation, adjusting for the young person's sex and the mother's age, highest qualification, occupational social class, and employment status. We then used Stata's *paramed* (Emsley and Liu, 2013) to conduct counterfactual mediation analysis. This approach, unlike traditional mediation analysis, generates estimates that have precise interpretations in terms of counterfactuals. For each mediator, we estimated the indirect effect (via the mediator), the direct effect (not via the mediator), and the proportion of the total effect mediated. Mediation models were also adjusted for the mother's age, highest qualification, occupational social class, and employment status, and assumed no interaction effect between the exposure and mediator. Linear regression was used to estimate associations between maternal depressive symptoms and the distance to the young person's university. Logistic regression was used to estimate associations with reasons for choosing a particular university or choosing not to attend university. These analyses adjusted for the same covariates as the main models. Results based on complete-case data are included within the supplementary information. In further sensitivity analyses, we repeated analyses using two alternative versions of the exposure: the mother's raw EPDS score when the young person was aged 11, and the mother's raw EPDS score when the young person was aged 18. All analysis was carried out using Stata v17.

## 3 Results

### 3.1 Comparison of excluded and retained participants

A comparison of excluded and retained participants is provided in Supplementary Results and Supplementary Table S2. *t*-tests and chisquared tests using complete-case data showed that young people included in the analytic sample (*N* = 8952) were slightly less likely than excluded participants to report at age 26 having studied at university (64.3 % vs 71.4 %, *p* = 0.001). Mothers in the analytic sample were less socioeconomically advantaged than excluded mothers (e.g., 12.0 % vs 17.0 % with a degree, and 5.1 vs 8.9 % with a professional occupation). This likely reflects lower coverage by the NPD of private schools: 4.3 % of participants with available NPD data were in private schools at age 16, compared to around 7 % nationally at the time (Green et al., 2012).

### 3.2 Characteristics of the analytic sample

Table 1 shows sample descriptive characteristics of the analytic sample based on imputed data. Within the whole sample (N = 8952), 49.9 % of participants had studied at university by age 26, closely in line with the national participation rate of 47.0 % by age 25 for this cohort (Department for Education, 2023). 19.6 % of mothers scored 13+ on the EPDS once, and 15.7 % twice or more. Repeat measures of mother's depressive symptoms were related, with a correlation of 0.39 between the earliest and the latest measurements. Pairwise correlations between all EPDS measurements are presented in Supplementary Table S3. Table 1 also shows descriptive characterstics based on imputed data for young people who attended university, and young people who did not. University attendees and non-attendees could not be formally compared using imputed data because separate imputation models were run for these subgroups. Nevertheless, participants who did not study at university appeared less socioeconomically advantaged than those who did (e.g., 5.0 % vs 23.8 % of mothers had a university degree) and their mothers had experienced more depressive symptoms (17.4 % vs 12.5 % of mothers scored 13+ on the EPDS twice or more). At age 16, young people who did not study at university had a more external locus of control (mean 3.9, S.D. 2.2 vs mean 2.8, S.D. 1.9), and substantially lower educational achievement (e.g., GCSE capped points score of mean 308.5 (S.D. 76.5) vs mean 393.2 (S.D. 49.6). Descriptive characterstics of the imputed sample are broadly similar to characteristics of the complete-case sample (Supplementary Table S4) but with imputation having recovered individuals from more disadvantaged backgrounds. For example, 15.7 % of mothers in the imputed sample scored 13+ on the EPDS twice or more, compared to 11.0 % based on complete-case data.

### 3.3 Analysis of higher education participation

Logistic regression was used to examine the association between maternal depressive symptoms and higher education attendance, adjusting for the child's sex and the mother's age, education, occupational social class, and employment status. For each occasion a mother scored above the clinical cut-point (13+) on the EPDS, a young person was 12 % less likely to study at higher education (OR: 0.88; 95 % CI = 0.82,0.94, *p* < 0.001), net of confounders. Table 2 presents results of the mediation analysis. For each mediator in turn, it shows the total effect, natural indirect effect (the path from maternal depression to higher education participation through the mediator) and controlled direct effects (the path from maternal depression to higher education participation that does not go through the mediator). The strongest evidence of mediation was seen for age 14 educational achievement (OR for indirect effect: 0.90, CI = 0.88,0.93, *p* < 0.001) and age 16 educational achievement (OR for indirect effect: 0.84, CI = 0.80,0.88, p < 0.001). In both cases, a substantial proportion of the total effect was mediated by the indirect effect (respectively, 60.4 %, 82.7 %). In contrast, there was little evidence of mediation by the young person's own depressive symptoms at age 16 (indirect OR: 0.99, CI = 0.98,1.00, *p* = 0.12). There was evidence of mediation by locus of control at 16 (indirect OR: 0.95; 95 % CI = 0.94,0.97, p < 0.001), although here mediation was more modest than for educational achievement (34.4 % mediated).

### 3.4 Analysis of distance to university and reasons for choices around university

Among participants who studied at university (Table 3), there was little evidence that maternal depressive symptoms impacted the distance to university (for each additional time a mother scored 13 + on the EPDS, beta: 0.01 km, CI = −5.18,5.20, *p* = 0.99). There was little evidence of an impact on perceived importance of distance from family home (OR: 0.94, CI = 0.85,1.04, *p* = 0.23). However, maternal depressive symptoms were associated with wanting to stay close to family/children (OR: 1.18, CI = 1.03,1.35, *p* = 0.02). Among participants who did not attend university (Table 3), those whose mothers had reported elevated depressive symptoms were not more likely to have had concerns about being a financial burden (for each additional time a mother scored 13 + on the EPDS, OR:1.08, CI = 0.95,1.22, *p* = 0.24). However, they were more likely to give as a reason having had other priorities relating to family or children (OR: 1.17, CI = 1.02,1.35, *p* = 0.03).

### 3.5 Sensitivity analyses

Results based on complete-case data (Supplementary Tables S5-S6) were consistent with main results, although much less precise due to the smaller sample sizes (*N* = 1408 for mediation analysis, *N* = 991 for outcomes specific to university attendees, *N* = 389 for outcomes specific to non-attendees). Associations with the mother's raw EPDS scores when the young person was aged 11 and 18 were qualitatively similar to the main results (Supplementary Tables S7-S8).

## 4 Discussion

This study examined the impact of elevated maternal depressive symptoms throughout childhood and adolescence on young people's higher education participation. We investigated the mediating role played by the young person's own depressive symptoms at 16, educational achievement at age 14 and age 16, and locus of control at 16. We also explored the reasons why young people whose mothers had experienced elevated depressive symptoms who participate in higher education choose the university they do, and the reasons given by others for not attending university.

Logistic regression found that for each time a mother had experienced clinically elevated depressive symptoms out of five measurement occasions during the young person's lifetime, the young person was 12 % less likely to study at university. Since all models adjusted for confounders including maternal education and social class, this is unlikely to simply reflect socio-economic differences which independently affect the mother's mental health and the child's educational outcomes. Mediation analysis found that the association between maternal depressive symptoms and higher education participation was largely explained by the young person's educational achievement in secondary school. This suggests that the impact of maternal depression during the child's lifetime on continuation to higher education is in large part due to earlier effects on academic attainment. It should be noted that we considered maternal depressive symptoms across the child's lifetime from one to 18 years (Brophy et al., 2021; Shen et al., 2016). Previous studies suggest that maternal depression more strongly influences children's academic attainment when maternal depression occurs during the child's lifetime, rather than prior to their birth, and when it is more recurrent (Brophy et al., 2021). Evidence also suggests stronger effects of maternal rather than paternal depression on children's academic attainment (Brophy et al., 2021; Shen et al., 2016). Our results are also in line with research indicating that maternal depression can negatively impact educational outcomes throughout secondary school (Pearson et al., 2016; Psychogiou et al., 2020). This study builds on those findings, exploring longer-term impacts by looking at higher education participation. Sensitivity analyses found similar relationships when using the mother's raw EPDS score when the child was aged 11 or when they were 18. This is consistent with stability of maternal depressive symptoms across childhood and adolescence (Hammerton et al., 2015).

Results suggested only a modest role of the young person's own depressive symptoms in explaining the link between maternal depressive symptoms and higher education participation. This suggests a focus purely on young people's mental health will not alleviate educational inequalities for children whose parents have mental health problems. We were not able to investigate the role of potentially important parenting behaviours, for example parental warmth and interest (Lovejoy et al., 2000) or involvement in stimulating activities. This has been associated with cognitive resilience in children (Kim-Cohen et al., 2004), and may be impacted by parental depression along with other aspects of functioning (Lewis et al., 2013). In contrast, there was some evidence of mediation by the young person's locus of control at 16, which was more external for those who had experienced maternal depression. This is consistent with a body of work from longitudinal studies and intervention studies highlighting the importance of a range of related individual cognitive variables such as locus of control and self-efficacy in explaining outcomes following a range of adverse experiences (Buddelmeyer and Powdthavee, 2016; Collishaw et al., 2016; Ng-Knight and Schoon, 2017). Further exploration of factors that underlie development of greater self-efficacy or a more internal locus of control (Nowicki et al., 2018), particularly in vulnerable populations, is warranted. There are also parallels between locus of control and academic self-efficacy, which is linked with academic attainment (Honicke and Broadbent, 2016).

Among university attendees, those whose mothers had clinically elevated depressive symptoms on more occasions did not differ in the distance travelled to university, but a desire to stay close to family or children was more often cited as important for their choice of institution. Meanwhile, among young people who chose not to study at university, those whose mothers had clinically elevated depressive symptoms on more occasions more often reported that having other priorities to do with family or children had been a consideration. The phrasing of questions means that in both cases it is unclear which family member(s) respondents had in mind, limiting the conclusions which can be drawn. However, results are consistent with an influence of perceived family responsibility both on the decision to participate in higher education and on choice of institution for young people affected by maternal depression. Sensitivity analyses considering maternal depressive symptoms when the young person was aged 11 and 18 years gave broadly similar results, suggesting that the pattern of maternal depression symptoms throughout a child's lifetime may be relevant to these decisions.

A major strength is the follow-up of mothers and their children across childhood and adolescence into adulthood, which meant long-term impacts of maternal depressive symptoms on academic attainment could be explored. Rich longitudinal data meant we were able to investigate potential mediators between maternal depressive symptoms and young people's higher education participation, whilst controlling for important socioeconomic confounders. Although socioeconomic differences in loss to follow-up in this cohort have been noted (Howe et al., 2013), our multiply imputed sample was representative of the target population on key characteristics: 49.9 % of participants studied at university, compared to around 47.0 % nationally for this cohort (Department for Education, 2023). Thus, multiple imputation is likely to have partly recovered the original distributions of key characteristics, reducing the impact of attrition. Nevertheless, the study had limitations relating to the nature of the study sample, measurement of constructs, and identification of causal processes. Loss to follow-up reduced the sample size, and some variables had a high rate of imputation. Selective non-response by maternal depression is plausible, and imputation may not have accurately corrected for this. As in any observational study, some residual confounding will remain, and confounding factors will not have been fully corrected for where there is measurement error in covariates. Depressive symptoms were based on self-report data, and results based on a clinical assessment may have differed. Information was not available on treatment of maternal depression, which may impact on outcomes for young people (Cuijpers et al., 2015; Wickramaratne et al., 2011). The main exposure considered maternal depressive symptoms at five timepoints including when the young person was aged 18, slightly after measurement of the mediators. To the extent that these mediators may have influenced this last measure of maternal depressive symptoms, results of mediation models could be biased. However, sensitivity analyses using the last available measure of maternal depressive symptoms before the mediators gave similar results. Information was not available on co-parental depression, which could co-act with maternal depression to influence young people's choices, and we could not examine other possible mediators including parenting behaviour. While educational attainment in secondary school emerged as a key mediator, further research will be needed to understand how this is impacted by maternal depression. Lack of income measures meant we could not explore mediation via household income, a dynamic aspect of socioeconomic position whose associations with maternal mental health are likely bidirectional (Thomson et al., 2022). Follow-up work could explore this in surveys with annually repeated income measures (Akanni et al., 2022). We considered mediators individually, but these factors may act jointly to influence young people's educational outcomes, such that the overall role of these mediators would be less than the sum of their individual indirect effects. Follow-up work could use approaches which can examine multiple mediators simultaneously, to further unpick these relationships. For higher education participation, we considered whether young people had studied at university by age 26 but not later in life, and rates of drop-out may also differ among young people affected by maternal depression. Although we were able to investigate participants’ reasons for their choice of university, a full exploration of undermatching would require data on how well-matched entry requirements for individual courses were to participants’ educational achievement at 18.

### 4.1 Implications

The finding that young people whose mothers have experienced more depressive symptoms during their lifetime are less likely to study at higher education is noteworthy, considering the impact that achieving a degree can have on future earnings across a person's lifetime (Belfield et al., 2020; Britton et al., 2020). Moreover, results pointed to educational achievement early in secondary school as an important mediator of this relationship, suggesting low educational attainment at this age may function as a developmental ‘snare’ which can lead to continued disadvantage (Moffitt, 1993). Interventions for young people to improve educational outcomes may wish to focus on improving earlier educational outcomes, ideally before or at the beginning of secondary school as this may have the best chance of improving their attainment. Exploring influences on cognitive variables such locus of control, which we also identified as a mediator, will be important for understanding how best to support academic attainment, as well as good mental health (Collishaw et al., 2016), in this vulnerable population. At the same time, intervention to help with maternal depression itself remains important, both for the mother and for her children. Previous evidence suggests pharmacological and psychological treatments can reduce the symptoms of depression in mothers, as well as improving the wellbeing of her children (Cuijpers et al., 2015; Wickramaratne et al., 2011).

## 5 Conclusion

Young people whose mothers had experienced clinically elevated depressive symptoms on more occasions were considerably less likely to study at university. Of the mediators explored, the most important was educational achievement during secondary school. Results therefore highlight the importance of academic attainment at school for promoting life chances in young people from families affected by maternal depression.

## Supplementary Material

Supplementary data to this article can be found online at https://doi.org/10.1016/j.jad.2023.10.061.

Supplementary material

## Figures and Tables

**Table 1:** Descriptive statistics of all variables included in the analysis, imputed data (*n* = 8952).

		All participants *N*= 8952^a^	Attended university *N* = 2037	Did not attend university *N* = 1129
Continuous variables		Mean (SD)	Mean (SD)	Mean (SD)
Age of mother at birth		28.3 (4.7)	29.8 (4.3)	28.2 (4.7)
Maternal depressive symptoms: number of times mother scored 13+ on the Edinburgh Postnatal Depression Scale (EPDS)^b^		0.63 (1.1)	0.52 (1.0)	0.67 (1.1)
Young person's educational achievement at age 14^c^		107.0 (24.3)	122.1 (17.7)	102.6 (21.3)
Young person's educational achievement at age 16^d^		326.7 (90.2)	393.2 (49.6)	308.5 (76.5)
Young person's depressive symptoms at 16: 12-item Short Mood and Feelings Questionnaire (SMFQ)		5.8 (5.6)	5.8 (5.3)	6.5 (6.3)
Young person's locus of control at 16: 12-item Children's Nowicki- Strickland Internal-External scale (CNSIE)		3.5 (2.2)	2.8 (1.9)	3.9 (2.2)
Distance to university from family home (km)			121.9 (108.4)	
Categorical variables		%	%	%
Maternal depressive symptoms: number of times mother scored 13+ on the Edinburgh Postnatal Depression Scale (EPDS)^e^	0	64.8	68.9	63.5
	1	19.6	18.6	19.1
	2 or more	15.7	12.5	17.4
Young person studied at higher education	No	50.1		
	Yes	49.9		
Young person's sex	Male	50.2	34.3	34.1
	Female	49.8	65.7	65.9
Maternal occupational social class	Professional	4.5	8.4	2.0
	Managerial	29.2	40.1	26.6
	Skilled non- manual	45.2	40.0	49.3
	Skilled manual	8.3	4.6	9.1
	Semi-skilled	10.3	5.9	10.8
	Unskilled	2.5	0.9	2.2
Maternal highest qualification	Degree	11.7	23.8	5.0
	Vocational	9.9	5.5	12.8
	A level	23.0	32.1	20.5
	CSE/GCSE	51.1	36.6	56.8
	None	4.4	2.1	5.0
Mother in work or voluntary work when young person is 11	Yes	76.9	84.9	76.4
	No	33.1	15.1	23.6
Reason chose university: distance to family home		Not important^f^	43.3	
		Important^g^	56.7	
Reason chose university: to stay close to family/children at school		Not important^f^	87.6	
		Important^g^	12.4	
Reason didn’t go to university: didn’t want to be a financial burden		Not important^f^		66.1
		Important^g^		33.9
Reason didn’t go to university: had other priorities (e.g., family/ children)		Not important^f^		84.8
		Important^g^		15.2

a In analyses of higher education participation (N = 8952) the outcome was imputed for some participants. For analyses specific to university attendees (N = 2037) and non-attendees (*N* = 1139), we restricted based on higher education participation as reported in the age 26 questionnaire.

b Main exposure in analyses. Possible range: 0–5. Mothers completed the EPDS when the young person was: 1, 5, 8, 11 and 18 years old.

c Summary score based on results in English, Maths and Science, range 1–141.

d Summary score based on the pupil's best 8 subjects, range 0–540.

e Included for descriptive purposes only: analyses used a continuous measure of number of times the mother scored 13+.

f Collapsed from: extremely unimportant, unimportant, neither important nor unimportant.

g Collapsed from: extremely important, important.

**Table 2:** Mediation of the association between maternal depressive symptoms^a^ and whether young person studied at higher education by the young person's educational achievement, depressive symptoms, and locus of control (*n* = 9852).

*Mediator*		OR	CI	p	% mediated
Educational achievement at 14	Total effect^b^	0.84	0.78,0.92	p < 0.001	60.4
	Controlled direct effect^c^	0.93	0.87,1.01	*p* = 0.08	
	Natural indirect effect^d^	0.90	0.88,0.93	p < 0.001	
Educational achievement at 16	Total effect	0.81	0.73,0.89	p < 0.001	82.7
	Controlled direct effect	0.96	0.89,1.05	*p* = 0.39	
	Natural indirect effect	0.84	0.80,0.88	p < 0.001	
Young person's depressive symptoms at 16	Total effect	0.88	0.82,0.94	p < 0.001	6.0
	Controlled direct effect	0.88	0.83-0.95	p < 0.001	
	Natural indirect effect	0.99	0.98-1.00	p = 0.12	
Young person's locus of control at 16	Total effect	0.87	0.82-0.94	p < 0.001	34.4
	Controlled direct effect	0.92	0.85-0.98	p = 0.02	
	Natural indirect effect	0.95	0.94,0.97	p < 0.001	

a Exposure: number of times the mother scored 13+ on the Edinburgh Postnatal Depression Scale during the child's lifetime. Odds ratios therefore represent associations per additional occasion.

b Total effect is the effect of the exposure on the outcome. Note that paramed calculates this from the direct and indirect effects, so the estimated total effect differs slightly between models.

c Controlled direct effect is the effect of the exposure on the outcome while controlling for the mediator.

d Natural indirect effect and is the effect of the exposure on the outcome that works through the mediator.

**Table 3:** Associations with maternal depressive symptoms^a^: outcomes specific to university attendees and non-attendees.

Young people who attended university (*N* = 2237)			
*Distance moved for university:*	Beta	CI	p
Distance from family home (km)	0.01	− 5.18,5.20	0.99
*Reasons why chose that university:*	OR^b^	CI	p
Important or extremely important: distance from family home	0.94	0.85,1.04	0.23
Important or extremely important: staying close to family/children	1.18	1.03,1.35	0.02
Young people who didn’t attend university (N = 1129)			
*Reasons why didn’t attend university:*	OR^b^	CI	p
Didn’t want to be a financial burden	1.08	0.95,1.22	0.24
Had other priorities (family/children)	1.17	1.02,1.35	0.03

a Exposure: number of times the mother scored 13+ on the Edinburgh Postnatal Depression Scale during the child's lifetime. Odds ratios therefore represent associations per additional occasion.

b ORs for rating this consideration as ‘important’ or ‘extremely important’.
