# Supplementary material for: Maternal depressive symptoms and young people's higher education participation and choice of university: Evidence from a longitudinal cohort study
Source: J Affect Disord. Author manuscript; Available in PMC 2025 Aug 18. (PMC7618021; doi:10.1016/j.jad.2023.10.061)
Supplement: Supplementary material [file EMS207921-supplement-Supplementary_material.docx]

**Supplementary material:**

***Supplementary methods:***

Description of the cohort

Multiple imputation

***Supplementary results:***

Comparison of excluded and retained participants

***Figures:***

Figure S1: STROBE diagram showing exclusions from the analytic sample

***Tables:***

Table S1: Imputed data for analytic sample (N=8,952)

Table S2: Comparison of excluded participants (N=3,874), and participants retained in the analytic sample (N=8,952), based on complete-case data

Table S3: Correlation of maternal depressive symptoms across time, based on imputed data

(N=8,952)

Table S4: Descriptive characteristics of analytic sample (N=8,952), complete-case data

Table S5: Results of mediation analysis, complete-case analysis (N=1,408)

Table S6: Outcomes specific to attendees and non-attendees, complete-case analysis

Table S7: Mediation analysis, alternative specifications of maternal depressive symptoms (N=8,952)

Table S8: Outcomes specific to attendees and non-attendees, alternative specifications of maternal depressive symptoms (N=8,952)

***Supplementary methods:***

**Description of the cohort**

This analysis used data from the Avon Longitudinal Study of Parents and Children (ALSPAC), a pregnancy study of women with expected delivery dates between 1/4/1991 and 31/12/1992 who were living in or around Bristol (Boyd et al., 2013; Fraser et al., 2013; Northstone et al., 2019). From the initial 14,541 pregnancies, 13,988 children were alive at 1 year. When the oldest children were around 7 years old, the study enrolled 913 additional children who had met the original eligibility criteria but had not been initially recruited. The total sample size for analyses using data collected after the age of seven is therefore 15,447 pregnancies, from which 14,901 children were alive at 1 year of age. The mothers, children, and mothers’ partners have been followed up through regular questionnaires and clinic assessments collecting data on biological, environmental, and lifestyle factors (Boyd et al., 2013). Ethical approval for the study was obtained from the ALSPAC Ethics and Law Committee and the Local Research Ethics Committee. Informed consent for the use of data collected via questionnaires and clinics was obtained from participants following the recommendations of the ALSPAC Ethics and Law Committee at the time. At age 18, study children were sent 'fair processing' materials describing ALSPAC’s intended use of their health and administrative records and given clear means to consent or object via a written form. Data were not extracted for participants who objected, or who were not sent fair processing materials. Data from age 26 was collected using REDCap (Research Electronic Data Capture)(Harris et al., 2009). REDCap is a secure, web-based software platform designed to support data capture for research studies hosted at the University of Bristol.

**Multiple imputation**

Imputation models included all exposures, outcomes, mediators, and confounders used in analysis, with auxiliary variables including earlier measures of the young person’s depressive symptoms and locus of control. The mother’s continuous EPDS scores from when the young person was one, 5, 8, 11, and 18 years old were imputed as separate variables, and the main exposure (the number of occasions on which the mother had an EPDS score of 13+) was derived from these variables post-imputation. Continuous variables were imputed using predictive mean matching, binary variables with logistic regression, and categorical variables with ordered or multinomial logistic regression. The % of each variable which was imputed is shown in Table S3.

***Supplementary results:***

Comparison of included and excluded participants based on complete-case data

A comparison of T-tests and chi-squared tests using complete-case data (Supplementary Table S1) showed that young people included in the analytic sample (N=8,952) were slightly less likely than excluded participants (N=3,874) to report at age 26 having studied at university (64.3% vs 71.4%, p=0.001). Mothers in the analytic sample were less socioeconomically advantaged than excluded mothers both in terms of qualifications (12.0% vs 17.0% with a degree) and social class (5.1 vs 8.9% with a professional occupation). This is likely to reflect lower coverage by the NPD of private schools: 4.3% of all participants with available NPD data were in private schools at age 16, compared to around 7% nationally at the time (Green et al., 2012). On the other hand, retained young people had higher educational achievement at 14 and 16 (respectively, 106.1 vs 92.3 and 328.0 vs 279.0, both p<0.001). They did not differ in terms of gender, depressive symptoms at 16, or locus of control at 16. Retained mothers were older (mean age at the child’s birth: 28.3 vs 27.1, p<0.001). They did not differ on the number of times they had scored 13+ on the EPDS, but retained mothers were more likely to be have been in work when the child was aged 11 (79.0 vs 73.2%, p<0.001).

Figure S1: STROBE diagram showing exclusions from the analytic sample


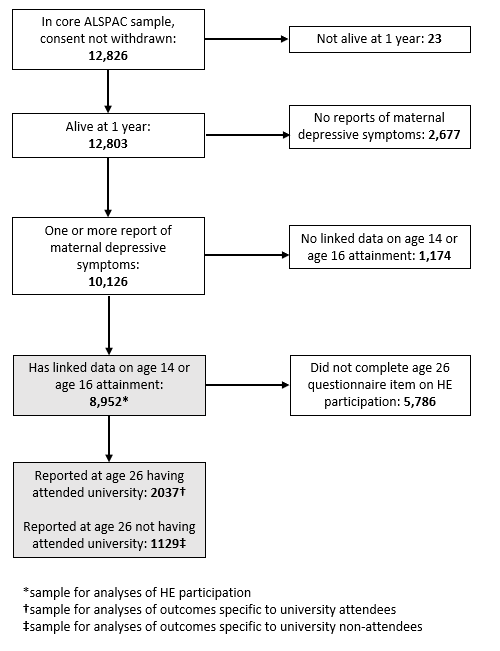


**Table S1: Imputed data for analytic sample (N=8952)**

| **Variable** | **imputed** | **% imputed** |
| --- | --- | --- |
| Young person’s sex | 0 | 0.0 |
| Maternal age | 304 | 3.4 |
| Maternal highest qualification | 683 | 7.6 |
| Maternal occupational social class | 2082 | 23.3 |
| Mum in work when young person aged 11 | 2862 | 32.0 |
| Maternal EPDS when young person aged 1 | 1107 | 12.4 |
| Maternal EPDS when young person aged 5 | 1986 | 22.2 |
| Maternal EPDS when young person aged 8 | 2796 | 31.2 |
| Maternal EPDS when young person aged 11 | 2879 | 32.2 |
| Maternal EPDS when young person aged 18 | 5594 | 62.5 |
| Young person’s higher education attendance | 5786 | 64.6 |
| Young person’s depressive symptoms at age 16 | 4927 | 55.0 |
| Young person’s locus of control at age 16 | 4998 | 55.8 |
| Young person’s educational achievement at age 14 | 94 | 1.1 |
| Young person’s educational achievement at age 16 | 807 | 9.0 |

| Table S2: Comparison of excluded participants (N=3874), and participants retained in the analytic sample (N=8952), based on complete-case data | | | | | | |
| --- | --- | --- | --- | --- | --- | --- |
|  | | **Excluded**  **N=3874** | | **Retained**  **N=8,952** | |  |
| Continuous variables | | **Mean (SD)** | **N** | **Mean (SD)** | **N** | **P for difference** |
| Age of mother at birth | | 27.1(5.3) | 3351 | 28.3(4.7) | 8648 | <0.001 |
| Maternal depressive symptoms: number of times mother scored 13+ on the Edinburgh Postnatal Depression Scale (EPDS)† | | 0.5 (1.0) | 624 | 0.5(1.0) | 4893 | 0.87 |
| Young person’s educational achievement at age 14‡ | | 92.3(28.1) | 1919 | 106.1(24.2) | 8145 | <0.001 |
| Young person’s educational achievement at age 16^§^ | | 279.0(104.1) | 2000 | 328.0(89.1) | 8858 | <0.001 |
| Young person’s depressive symptoms at 16: 12-item Short Mood and Feelings Questionnaire (SMFQ) | | 5.9(5.5) | 673 | 5.9(5.6) | 4025 | 0.90 |
| Young person’s locus of control at 16: 12-item Children's Nowicki-Strickland Internal-External scale (CNSIE) | | 3.1(2.0) | 744 | 3.2(2.1) | 3954 | 0.09 |
| Categorical variables | | **%** | **N** | **%** | **N** | **P for difference** |
| Maternal depressive symptoms: number of times mother scored 13+ on the Edinburgh Postnatal Depression Scale (EPDS)† | 0 | 71.8 | 624 | 70.4 | 4893 | 0.71 |
|  | 1 | 17.3 |  | 18.6 |  |  |
|  | 2 or more | 10.9 |  | 11.0 |  |  |
| Young person studied at higher education | No | 28.6 | 660 | 35.7 | 3166 | 0.001 |
|  | Yes | 71.4 |  | 64.3 |  |  |
| Young Person’s sex | Male | 50.4 | 3851 | 50.2 | 8952 | 0.82 |
|  | Female | 49.6 |  | 49.8 |  |  |
| Maternal occupational social class | Professional | 8.9 | 1959 | 5.1 | 6870 | <0.001 |
|  | Managerial | 34.7 |  | 31.1 |  |  |
|  | Skilled non-manual | 35.4 |  | 44.5 |  |  |
|  | Skilled manual | 7.5 |  | 7.8 |  |  |
|  | Semi-skilled | 11.0 |  | 9.4 |  |  |
|  | Unskilled | 2.5 |  | 2.2 |  |  |
| Maternal highest qualification | Degree | 17.0 | 2537 | 12.0 | 8269 | <0.001 |
|  | Vocational | 9.4 |  | 9.8 |  |  |
|  | A Level | 21.0 |  | 23.1 |  |  |
|  | CSE/GCSE | 46.0 |  | 50.8 |  |  |
|  | None | 5.8 |  | 4.3 |  |  |
| Mother in work or voluntary work when young person is 11 | Yes | 73.2 | 721 | 79.0 | 6090 | <0.001 |
|  | No | 26.8 |  | 21.0 |  |  |
| *Descriptive characteristics based on imputed data are shown in Table 1.  ^†^Possible range: 0-5. Mothers completed the EPDS when the young person was: 1, 5, 8, 11 and 18 years old.  ^‡^Summary score based on results in English, Maths and Science, range 1-141  ^§^Summary score based on the pupil’s best 8 subjects, range 0-540  ^‖^Collapsed from: extremely unimportant, unimportant, neither important nor unimportant. ^¶^Collapsed from: extremely important, important. | | | | | | |

**Table S3: Correlation of maternal depressive symptoms across time, based on imputed data (N=8,952)***

|  | **1 year** | **5 years** | **8 years** | **11 years** | **18 years** |
| --- | --- | --- | --- | --- | --- |
| **1 year** | 1.00 |  |  |  |  |
| **5 years** | 0.46 | 1.00 |  |  |  |
| **8 years** | 0.43 | 0.49 | 1.00 |  |  |
| **11 years** | 0.41 | 0.46 | 0.49 | 1.00 |  |
| **18 years** | 0.39 | 0.42 | 0.39 | 0.45 | 1.00 |
| *Correlations within all participants | | | | | |

| Table S4: Descriptive characteristics of analytic sample (N=8952), complete-case data | | | | | | | |
| --- | --- | --- | --- | --- | --- | --- | --- |
|  | | **All participants**  **N=8,952** | | **Attended university**  **N=2,037** | | **Did not attend university**  **N=1,129** | |
| Continuous variables | | **Mean (SD)** | **N** | **Mean (SD)** | **N** | **Mean (SD)** | **N** |
| Age of mother at birth | | 28.3(4.7) | 8648 | 29.8(4.3) | 1957 | 28.2(4.7) | 1082 |
| Maternal depressive symptoms: number of times mother scored 13+ on the Edinburgh Postnatal Depression Scale (EPDS)† | | 0.5(1.0) | 4893 | 0.5(1.0) | 1538 | 0.6(1.0) | 749 |
| Young person’s educational achievement at age 14‡ | | 106.1(24.2) | 8145 | 121.4(17.9) | 1779 | 102.5(21.2) | 1042 |
| Young person’s educational achievement at age 16^§^ | | 328.0(89.1) | 8858 | 393.2(49.6) | 2034 | 308.8(76.2) | 1123 |
| Young person’s depressive symptoms at 16: 12-item Short Mood and Feelings Questionnaire (SMFQ) | | 5.9(5.6) | 4025 | 5.8(5.2) | 1643 | 6.6(6.3) | 727 |
| Young person’s locus of control at 16: 12-item Children's Nowicki-Strickland Internal-External scale (CNSIE) | | 3.2(2.1) | 3954 | 2.7(1.9) | 1622 | 3.8(2.2) | 706 |
| Distance to university from family home (km) | |  |  | 122.2(108.3) | 2009 |  |  |
| Categorical variables | | **%** | **N** | **%** | **N** | **%** | **N** |
| Maternal depressive symptoms: number of times mother scored 13+ on the Edinburgh Postnatal Depression Scale (EPDS)† | 0 | 70.4 | 4893 | 71.6 | 1538 | 70.0 | 749 |
|  | 1 | 18.6 |  | 18.2 |  | 17.0 |  |
|  | 2 or more | 11.0 |  | 10.2 |  | 13.1 |  |
| Young person studied at higher education | No | 35.7 | 3166 | 0.0 | 2037 | 100.0 | 1129 |
|  | Yes | 64.3 |  | 100.0 |  | 0.0 |  |
| Young Person’s sex | Male | 50.2 | 8952 | 34.3 | 2037 | 34.1 | 1129 |
|  | Female | 49.8 |  | 65.7 |  | 65.9 |  |
| Maternal occupational social class | Professional | 5.1 | 6870 | 8.7 | 1717 | 2.2 | 885 |
|  | Managerial | 31.1 |  | 40.8 |  | 27.9 |  |
|  | Skilled non-manual | 44.5 |  | 39.4 |  | 49.0 |  |
|  | Skilled manual | 7.8 |  | 4.4 |  | 8.6 |  |
|  | Semi-skilled | 9.4 |  | 5.8 |  | 10.4 |  |
|  | Unskilled | 2.2 |  | 0.9 |  | 1.9 |  |
| Maternal highest qualification | Degree | 12.0 | 8269 | 23.9 |  | 5.1 | 1050 |
|  | Vocational | 9.8 |  | 5.4 |  | 12.7 |  |
|  | A Level | 23.2 |  | 32.2 |  | 20.7 |  |
|  | CSE/GCSE | 50.8 |  | 36.6 |  | 56.8 |  |
|  | None | 4.3 |  | 1.9 |  | 4.9 |  |
| Mother in work or voluntary work when young person is 11 | Yes | 79.0 | 6090 | 85.1 |  | 77.3 | 891 |
|  | No | 21.0 |  | 14.9 |  | 22.7 |  |
| Reason chose university: distance to family home | | Not important^‖^ |  | 43.3 | 2016 |  |  |
|  |  | Important^¶^ |  | 56.7 |  |  |  |
| Reason chose university: to stay close to family/children at school | | Not important^‖^ |  | 87.6 | 2008 |  |  |
|  |  | Important^¶^ |  | 12.4 |  |  |  |
| Reason didn’t go to university: didn’t want to be a financial burden | | Not important^‖^ |  |  |  | 66.1 | 1104 |
|  |  | Important^¶^ |  |  |  | 33.9 |  |
| Reason didn’t go to university: had other priorities (e.g., family/children) | | Not important^‖^ |  |  |  | 84.9 | 1103 |
|  |  | Important^¶^ |  |  |  | 15.1 |  |
| *Descriptive characteristics based on imputed data are shown in Table 1.  ^†^Possible range: 0-5. Mothers completed the EPDS when the young person was: 1, 5, 8, 11 and 18 years old.  ^‡S^ummary score based on results in English, Maths and Science, range 1-141  ^§^Summary score based on the pupil’s best 8 subjects, range 0-540  ^‖^Collapsed from: extremely unimportant, unimportant, neither important nor unimportant. ^¶^Collapsed from: extremely important, important. | | | | | | | |

**Table S5: Results of mediation analysis, complete-case (N=1,408)**

| ***Mediator*** |  | **OR** | **CI** | **p** | **% mediated** |
| --- | --- | --- | --- | --- | --- |
| Educational achievement at 14 | Total effect* | 0.93 | 0.81,1.07 | 0.32 | 72.0 |
|  | Controlled direct effect^†^ | 0.98 | 0.86,1.12 | 0.77 |  |
|  | Natural indirect effect^‡^ | 0.95 | 0.90,1.00 | 0.05 |  |
| Educational achievement at 16 | Total effect | 0.93 | 0.79,1.10 | 0.41 | 115.7 |
|  | Controlled direct effect | 1.01 | 0.87,1.17 | 0.88 |  |
|  | Natural indirect effect | 0.92 | 0.84, 1.01 | 0.08 |  |
| Young person’s depressive symptoms at 16 | Total effect | 0.93 | 0.83,1.06 | 0.28 | 10.2 |
|  | Controlled direct effect | 0.94 | 0.83,1.06 | 0.33 |  |
|  | Natural indirect effect | 0.99 | 0.98,1.01 | 0.30 |  |
| Young person’s locus of control at 16 | Total effect | 0.93 | 0.82,1.06 | 0.27 | 41.9 |
|  | Controlled direct effect | 0.96 | 0.85,1.09 | 0.52 |  |
|  | Natural indirect effect | 0.97 | 0.95,0.99 | 0.009 |  |
| *Total effect is the effect of the exposure on the outcome. Note that paramed calculates this from the direct and indirect effects, so the estimated total effect differs slightly between models.  ^†^Controlled direct effect is the effect of the exposure on the outcome while controlling for the mediator.  ^‡^Natural indirect effect and is the effect of the exposure on the outcome that works through the mediator. | | | | | |

**Table S6: Outcomes specific to attendees and non-attendees, complete-case analysis**

| **Young people who attended university (N=991)** |  |  |  |
| --- | --- | --- | --- |
| *Distance moved for university:* | Beta | CI | p |
| Distance from family home (km) | 3.22 | -3.57,10.03 | 0.35 |
| *Reasons why chose that university:* | OR* | CI | p |
| Important or extremely important: distance from family home | 0.87 | 0.76,1.01 | 0.06 |
| Important or extremely important: staying close to family/children | 1.09 | 0.89,1.33 | 0.41 |
| **Young people who didn’t attend university (N=389)** |  |  |  |
| *Reasons why didn’t attend university:* | OR* | CI | p |
| Didn’t want to be a financial burden | 1.17 | 0.96,1.44 | 0.13 |
| Had other priorities (family/children) | 0.96 | 0.71,1.29 | 0.77 |
| *ORs for rating this consideration as ‘important’ or ‘extremely important’ | | | |

**Table S7: Mediation analysis, alternative specifications of maternal depressive symptoms**

| ***Exposure: mother’s raw EPDS score when young person aged 11*** | | | | | |
| --- | --- | --- | --- | --- | --- |
| **Mediator** |  | **OR** | **CI** | **p** | **% mediated** |
| Educational achievement at 14 | Total effect* | 0.97 | 0.95,0.99 | 0.004 | 80.8 |
|  | Controlled direct effect^†^ | 0.99 | 0.97,1.01 | 0.57 |  |
|  | Natural indirect effect^‡^ | 0.98 | 0.97,0.98 | <0.001 |  |
| Educational achievement at 16 | Total effect | 0.96 | 0.94,0.99 | 0.004 | 91.7 |
|  | Controlled direct effect | 1.00 | 0.97,1.02 | 0.78 |  |
|  | Natural indirect effect | 0.97 | 0.95,0.98 | <0.001 |  |
| Young person’s depressive symptoms at 16 | Total effect | 0.98 | 0.96,0.99 | 0.009 | 8.6 |
|  | Controlled direct effect | 0.98 | 0.96,1.00 | 0.02 |  |
|  | Natural indirect effect | 1.00 | 1.00,1.00 | 0.09 |  |
| Young person’s locus of control at 16 | Total effect | 0.98 | 0.96,0.99 | 0.01 | 42.4 |
|  | Controlled direct effect | 0.99 | 0.97,1.00 | 0.15 |  |
|  | Natural indirect effect | 0.99 | 0.99,0.99 | <0.001 |  |
| ***Exposure: mother’s raw EPDS score when young person aged 18*** | | | | | |
| **Mediator** |  | **OR** | **CI** | **p** | **% mediated** |
| Educational achievement at 14 | Total effect* | 0.97 | 0.95,1.00 | 0.02 | 70.8 |
|  | Controlled direct effect^†^ | 0.99 | 0.97,1.01 | 0.45 |  |
|  | Natural indirect effect^‡^ | 0.98 | 0.97,0.99 | <0.001 |  |
| Educational achievement at 16 | Total effect | 0.96 | 0.93,0.99 | 0.006 | 104.6 |
|  | Controlled direct effect | 1.00 | 0.98,1.03 | 0.89 |  |
|  | Natural indirect effect | 0.96 | 0.94,0.98 | <0.001 |  |
| Young person’s depressive symptoms at 16 | Total effect | 0.98 | 0.96,1.00 | 0.02 | 9.1 |
|  | Controlled direct effect | 0.98 | 0.96,1.00 | 0.04 |  |
|  | Natural indirect effect | 1.00 | 1.00,1.00 | 0.09 |  |
| Young person’s locus of control at 16 | Total effect | 0.98 | 0.96,1.00 | 0.02 | 53.8 |
|  | Controlled direct effect | 0.99 | 0.97,1.01 | 0.31 |  |
|  | Natural indirect effect | 0.99 | 0.98,0.99 | <0.001 |  |
| *Total effect is the effect of the exposure on the outcome. Note that paramed calculates this from the direct and indirect effects, so the estimated total effect differs slightly between models.  ^†^Controlled direct effect is the effect of the exposure on the outcome while controlling for the mediator.  ^‡^Natural indirect effect and is the effect of the exposure on the outcome that works through the mediator. | | | | | |

**Table S8: Outcomes specific to attendees and non-attendees, alternative specifications of maternal depressive symptoms**

| ***Exposure: mother’s raw EPDS score when young person aged 11*** | | | |
| --- | --- | --- | --- |
| **Young people who attended university (N=2,237)** |  |  |  |
| *Distance moved for university:* | Beta | CI | p |
| Distance from family home (km) | 0.44 | -0.78,1.65 | 0.48 |
| *Reasons why chose that university:* | OR* | CI | p |
| Important or extremely important: distance from family home | 0.99 | 0.96,1.01 | 0.26 |
| Important or extremely important: staying close to family/children | 1.04 | 1.00,1.07 | 0.03 |
| **Young people who didn’t attend university (N=1,129)** |  |  |  |
| *Reasons why didn’t attend university:* | OR* | CI | p |
| Didn’t want to be a financial burden | 1.02 | 0.99,1.06 | 0.17 |
| Had other priorities (family/children) | 1.02 | 0.98,1.06 | 0.32 |
| ***Exposure: mother’s raw EPDS score when young person aged 18*** | | | |
| **Young people who attended university (N=2,237)** |  |  |  |
| *Distance moved for university:* | Beta | CI | p |
| Distance from family home (km) | 0.74 | -0.59,2.07 | 0.28 |
| *Reasons why chose that university:* | OR* | CI | p |
| Important or extremely important: distance from family home | 1.00 | 0.97,1.02 | 0.73 |
| Important or extremely important: staying close to family/children | 1.05 | 1.01,1.09 | 0.009 |
| **Young people who didn’t attend university (N=1,129)** |  |  |  |
| *Reasons why didn’t attend university:* | OR* | CI | p |
| Didn’t want to be a financial burden | 0.99 | 0.96,1.03 | 0.68 |
| Had other priorities (family/children) | 1.05 | 1.00,1.10 | 0.04 |
| *ORs for rating this consideration as important or extremely important | | | |

Boyd, A., Golding, J., Macleod, J., Lawlor, D. A., Fraser, A., Henderson, J., Molloy, L., Ness, A., Ring, S., & Smith, G. D. (2013). Cohort profile: The ’Children of the 90s’-The index offspring of the avon longitudinal study of parents and children. *International Journal of Epidemiology*. https://doi.org/10.1093/ije/dys064

Fraser, A., Macdonald-wallis, C., Tilling, K., Boyd, A., Golding, J., Davey smith, G., Henderson, J., Macleod, J., Molloy, L., Ness, A., Ring, S., Nelson, S. M., & Lawlor, D. A. (2013). Cohort profile: The avon longitudinal study of parents and children: ALSPAC mothers cohort. *International Journal of Epidemiology*. https://doi.org/10.1093/ije/dys066

Green, F., Machin, S., Murphy, R., & Zhu, Y. (2012). The Changing Economic Advantage from Private Schools. *Economica*, *79*(316), 658–679. https://doi.org/https://doi.org/10.1111/j.1468-0335.2011.00908.x

Harris, P. A., Taylor, R., Thielke, R., Payne, J., Gonzalez, N., & Conde, J. G. (2009). Research electronic data capture (REDCap)—A metadata-driven methodology and workflow process for providing translational research informatics support. *Journal of Biomedical Informatics*, *42*(2), 377–381. https://doi.org/https://doi.org/10.1016/j.jbi.2008.08.010

Northstone, K., Lewcock, M., Groom, A., Boyd, A., Macleod, J., Timpson, N. J., & Wells, N. (2019). The Avon Longitudinal Study of Parents and Children (ALSPAC): an update on the enrolled sample of index children in 2019 [version 1; peer review: 2 approved]. *Wellcome Open Research*, *4*(51). https://doi.org/10.12688/wellcomeopenres.15132.1
